# Assessment of Knowledge and Practices of Medical Emergencies in Medical and Dental Students of the University of Ha'il, Saudi Arabia: A Cross-Sectional Study

**DOI:** 10.7759/cureus.70548

**Published:** 2024-09-30

**Authors:** Ibtihag S Elnaem, Albandri M Alghris, Layla H Alenzi, Hamdan A Alshammari, Rayan B Alanazi, Abdulaziz M Alshammari, Osama Y Alsalimi, Abdulkarim A ALnasrallah, Basal R Alkhalil, Lama W Albaker, Nawaf M Alharbi, Abdulsalam S Aldhmadi, Saleh A Aloqla, Bassam A Altayyar, Suliman M Aloqla, Abdullah M Alsubaie

**Affiliations:** 1 Oral and Maxilofacial Surgery, College of Dentistry, University of Ha'il, Ha'il, SAU; 2 Dentistry, College of Dentistry, University of Ha'il, Ha'il, SAU; 3 Medicine, College of Medicine, University of Ha'il, Ha'il, SAU; 4 Dentistry, King Khalid University, Abha, SAU

**Keywords:** assessment, saudi arabia, ha’il, medical student and dental students, medical emergency

## Abstract

Aim and background

Medical emergencies (MEs) pose urgent threats to life and occur frequently, with primary care providers seeing at least one emergency per year. Common MEs include asthma, anaphylaxis, seizures, and cardiac arrest. However, many medical offices are ill-equipped to handle these emergencies. Practices should obtain appropriate emergency equipment and medications based on their patient population, provider expertise, and proximity to the ER. A lack of emergency preparedness and training can have fatal consequences, so all healthcare providers must be well-prepared to respond. Providing basic life support (BLS) is the crucial first step until definitive treatment is available. The study aimed to assess emergency-related knowledge, its relationship to expertise, and the need for emergency management training among medical/dental students at University of Ha’il (UOH) in Saudi Arabia.

Methods

The study included 214 participants. It consisted of male and female dental and medical students in their clinical years at the UOH in Saudi Arabia. This targeted sampling approach ensured the study focused on the relevant demographic factors of dental and medical students to provide insights into their level of MR-related knowledge and its relationship to their expertise, as well as their awareness and need for training in ME management. The researchers aimed to assess these factors among the medical and dental students at the UOH.

Result

Dental and medical students show a strong grasp of emergency care fundamentals, with 78.6% familiar with BLS protocols. Most students correctly identify crucial actions, such as assessing airway, breathing, and circulation (ABCs), and immobilizing the head and neck for spinal injuries. Responses to emergencies include starting chest compressions, calling 911, and administering appropriate treatments for cardiovascular, respiratory, and neurological issues. Despite high competency levels, continuous education is vital to maintain and enhance their preparedness for emergencies.

Conclusions

Dental and medical students demonstrate a solid understanding of BLS, with 78.6% familiar with protocols and many identifying critical emergency actions. However, inconsistencies in responses highlight the need for ongoing education to improve decision-making and preparedness. Continuous training will ensure these future healthcare professionals are equipped to handle real-world emergencies effectively.

## Introduction

A medical emergency (ME) is defined as an acute injury or sickness that presents an immediate risk to an individual's life or long-term health. Certain emergencies, like heart, respiratory, and gastrointestinal problems, are too big for the victim to handle alone; therefore, they could need help from another trained individual. An ME is a disease or severe injury that poses an urgent threat to an individual's life [1]. It can occur at any moment. The incidence of MEs has indicated at least two crises every year [2]. Most primary-care physicians report at least one emergency presentation in their practice each year. The most common adult and childhood crises in the office include asthma, anaphylaxis, shock, convulsions, syncope, angina pectoris, postural hypertension, foreign object aspiration, hypoglycemia, and seizures [3,4]. Seizures and epilepsy consequences are a common reason for emergency medical examination, accounting for 5% of 911 calls and 1% of emergency room visits [5]. The dentist's expertise and readiness are crucial in handling such situations and providing treatment and patient care. By obtaining a complete patient history, doing a thorough examination, and occasionally altering treatment protocols, dentists can prevent up to 90% of medical catastrophes [5]. 

However, most offices are not adequately prepared to handle these medical situations. Practices can start a readiness programmed by obtaining emergency equipment and medications that match the range of potential emergencies in their patient populations, the practitioners' expertise, and the distance to the nearest emergency room [3]. A lack of training and skill to deal with MEs can have fatal implications as well as legal action. As a result, all health practitioners, including dentists, must be well prepared to respond to MEs. Giving basic life support (BLS) is the dentist's most important contribution until conclusive therapy for an ME becomes available [6]. The most effective way to deal with an emergency crisis is to thoroughly prepare for such a vital moment [7]. The purpose of this study was to assess the level of ME-related knowledge and its relationship to expertise, as well as the awareness and need for training in ME management among medical and dental students and trainers at the University of Ha'il (UOH) in Saudi Arabia.

## Materials and methods

Study design 

A cross-sectional study was conducted through online survey among dental and medical students at the UOH between July 2024 and August 2024.

Study criteria (inclusion and exclusion)

The study included male and female participants who were students at the UOH, enrolled in the clinical years of either their medical or dental programs. The inclusion criteria specified that participants must be studying medicine or dentistry and have reached the clinical stage of their education at the UOH. Conversely, the exclusion criteria excluded any individuals who were not students at the UOH or were not enrolled in the clinical years of their respective medical or dental programs.

Data collection

The estimated sample size for this study was determined to be 214 participants. This sample size was calculated using an online sample size calculator (Calculator.net), with a 95% confidence level and a 5% margin of error [8]. The population proportion rate was set at 50%, and the approximate population size was 480. The researchers opted for this sample size to ensure statistical rigor and the ability to draw meaningful conclusions from the data. The sampling technique employed in this study was convenience sampling. This non-probability sampling method allowed the researchers to collect data from readily available participants, in this case, medical and dental students enrolled at the UOH. The data collection tool utilized was an online self-response questionnaire, which was distributed to the target population through various web platforms. This approach facilitated the collection of data from a diverse range of medical and dental students at UOH.

Data analysis

The data collection process for this study involved the use of Google Forms, with the responses being stored in a Google Sheets spreadsheet. This streamlined approach leveraged popular and widely used digital tools, ensuring efficient data collection and organization. The raw data collected through the online questionnaire was then coded and processed using Microsoft Excel, before being transferred to SPSS software (version 28) for statistical analysis. By employing the SPSS platform, the research team was able to utilize advanced statistical techniques to generate meaningful insights from the collected data, addressing the study's objectives in a rigorous and professional manner. This multi-step data management strategy, utilizing a combination of Google Forms, Google Sheets, Microsoft Excel, and SPSS software, allowed the researchers to systematically collect, organize, and analyze the data, ultimately facilitating the generation of robust and reliable findings. 

## Results

Table 1 is based on the questionnaire responses from 214 male and female students in general medicine and dentistry.

**Table 1:** Demographic questions in the survey and responses

Questions	Choices	Frequency	Percentage	Valid Percentage	Cumulative Percentage
What is your gender?	Female	79	36.7	36.7	36.7
	Male	136	63.3	63.3	100.0
Which of the following best describes your current level of study in a dental or medical degree program?	4th-year dental student	28	13.0	13.0	13.0
	5th-year dental student	41	19.1	19.1	32.1
	6th-year dental student	19	8.8	8.8	40.9
	Dental intern	24	11.2	11.2	52.1
	4th-year medical student	23	10.7	10.7	62.8
	6th-year medical student	25	11.6	11.6	74.4
	Medical intern	36	16.7	16.7	91.2

In Table 2, we provide a comprehensive analysis of responses to BLS knowledge and emergency response capabilities among dental and medical students. This analysis is critical to understanding the readiness of these future healthcare professionals to effectively handle MEs. The results of the study reveal that a large majority of students (78.6%) reported that they were familiar with the proper steps to provide BLS during an ME. This high percentage indicates a generally strong basic knowledge among students regarding basic emergency-care procedures.

**Table 2:** The questionnaire and responses BLS: Basic life support; CPR: Cardiopulmonary resuscitation; ABCs: Airway, breathing, and circulation; EMS: Emergency medical services; MI: Myocardial infarction; ME: Medical emergency

Questions	Choices	Frequency	Percentage	Valid Percentage	Cumulative Percentage
BLS					
Do you know the appropriate steps to provide BLS during an ME until definitive care is available?	Yes	169	78.6	78.6	78.6
	No	46	21.4	21.4	100
When dealing with an unconscious patient, what should be your initial action?	Administer CPR immediately	11	5.1	5.1	5.1
	Check the patient’s ABCs	173	80.5	80.5	85.6
	Elevate the patient’s legs	9	4.2	4.2	89.8
	Look for identification to call emergency contacts	22	10.2	10.2	100
In the case of a suspected spinal injury, what is the top priority?	Move the patient to a more comfortable position	14	6.5	6.5	6.5
	Immobilize the patient’s head and neck	179	83.3	83.3	89.8
	Perform CPR if the patient is not breathing	9	4.2	4.2	94
	Check for other injuries before addressing the spine	13	6	6	100
A 45-year-old male collapses suddenly in a public place. On examination, he is unresponsive, not breathing normally, and has no pulse. What is the most appropriate initial step in managing this situation?	Start chest compressions	82	38.1	38.1	38.1
	Check for a pulse again	13	6	6	44.2
	Call for help and activate EMS	86	40	40	84.2
	Open the airway and check for breathing	34	15.8	15.8	100
Cardiovascular					
When treating a patient with a possible heart attack, what should be your first step?	Give the patient aspirin if they are not allergic	123	57.2	57.2	57.2
	Perform chest compressions	41	19.1	19.1	76.3
	Place the patient in a recovery position	45	20.9	20.9	97.2
	Encourage the patient to walk around	6	2.8	2.8	100
Which of the following is NOT a possible sign of MI?	Dyspnea	9	4.2	4.2	4.2
	Diaphoresis	35	16.3	16.3	20.5
	Chest pain	8	3.7	3.7	24.2
	Hemoptysis	163	75.8	75.8	100
A 30-year-old female presents with sudden onset of chest pain radiating to her left arm and jaw. She appears diaphoretic and anxious. What is the most likely diagnosis, and what immediate action should be taken?	MI; administer aspirin and call EMS	181	84.2	84.2	84.2
	Anxiety attack; reassurance and observation	26	12.1	12.1	96.3
	Pneumothorax; perform needle decompression	7	3.3	3.3	99.5
	Gastric reflux; administer antacid and observe	1	0.5	0.5	100
Which medication is commonly used to manage acute chest pain suspected to be from MI?	Metoprolol	30	14	14	14
	Nitroglycerin	143	66.5	66.5	80.5
	Atropine	19	8.8	8.8	89.3
	Digoxin	23	10.7	10.7	100
Respiratory					
What is the first-line treatment for acute asthma exacerbation in an emergency?	Intravenous corticosteroids	59	27.4	27.4	27.4
	Inhaled albuterol	122	56.7	56.7	84.2
	Oral prednisone	9	4.2	4.2	88.4
	Epinephrine	25	11.6	11.6	100
In the event of asthmatic shock, which of the following should be done?	Put patient upright	7	3.3	3.3	3.3
	Give the patient her or his usual inhaled brochodilator	19	8.8	8.8	12.1
	Consider nebulised salbutamol, supplementary oxygen or even subcutaneous epinephrine	19	8.8	8.8	20.9
	All of above	170	79.1	79.1	100
Which of the following should be done if you suspect that the patient has aspirated a foreign body?	If the patient is asymptomatic, there is no need to refer the patient for further management in the hospital	29	13.5	13.5	13.5
	Quickly put the suction into the patient's throat to suck out the foreign body	42	19.5	19.5	33
	Put the patient in reverse Trendelenburg position and encouraged coughing	110	51.2	51.2	84.2
	Ask the patient to check his or her stool to ensure that the foreign body has been passed out	34	15.8	15.8	100
Neurological disorders					
What is the first-line treatment for managing status epilepticus?	Diazepam intravenously	117	54.4	54.4	54.4
	Phenytoin intramuscularly	26	12.1	12.1	66.5
	Lorazepam intramuscularly	59	27.4	27.4	94
	Levetiracetam orally	13	6	6	100
Which of the following is the most appropriate immediate action for managing a suspected stroke in the clinic?	Administering aspirin	40	18.6	18.6	18.6
	Calling emergency services, monitoring vital signs, and providing supportive care.	156	72.6	72.6	91.2
	Giving the patient a glucose tablet and observing for improvement.	13	6	6	97.2
	Advising the patient to rest and scheduling a follow-up appointment with their physician.	6	2.8	2.8	100
Bleeding disorders					
What is the priority when managing a patient with severe bleeding?	Apply a splint to the affected limb	13	6	6	6
	Check for a pulse	21	9.8	9.8	15.8
	Apply direct pressure to the wound	180	83.7	83.7	99.5
	Give the patient water to drink	1	0.5	0.5	100
In case of hemorrhage, which of the following hemostatic agents can stop the bleeding immediately:	⁠Intravenous or intramuscular tranexamic acid	63	29.3	29.3	29.3
	⁠Tranexamic acid gel foam	52	24.2	24.2	53.5
	⁠Intravenous fibrinogen	61	28.4	28.4	81.9
	Gelatin-based such as Surgifoam and Floseal	39	18.1	18.1	100
Endocrine disorders					
A 55-year-old diabetic male complains of feeling dizzy and weak. His blood glucose level is 40 mg/dL (2.2 mmol/L). What is the immediate management for this patient?	Administer intravenous glucose	75	34.9	34.9	34.9
	Administer insulin	26	12.1	12.1	47
	Administer oral glucose	107	49.8	49.8	96.7
	Monitor closely without intervention	7	3.3	3.3	100
What is the recommended drug for immediate treatment of hypoglycaemia in a diabetic patient who is unconscious?	Oral glucose tablets	27	12.6	12.6	12.6
	Intravenous dextrose	148	68.8	68.8	81.4
	Subcutaneous insulin	26	12.1	12.1	93.5
	Oral sucrose solution	14	6.5	6.5	100
What is the most appropriate approach to managing an adrenal crisis in a clinical setting?	Administering intravenous hydrocortisone immediately	36	16.7	16.7	16.7
	Monitoring and correcting electrolyte imbalances	30	14	14	30.7
	Providing aggressive fluid resuscitation	12	5.6	5.6	36.3
	All of the above	137	63.7	63.7	100
How should dental and medical students manage the relationship between thyroid crisis and vasoconstrictors to avoid complications?	Avoid using vasoconstrictors in patients with a history of thyroid crisis	27	12.6	12.6	12.6
	Monitor vital signs closely during procedures involving vasoconstrictors	17	7.9	7.9	20.5
	Consult with an endocrinologist before administering vasoconstrictors	22	10.2	10.2	30.7
	All of the above	149	69.3	69.3	100
Overdose					
What is the initial treatment for a patient with suspected opioid overdose?	Administering naloxone intravenously	129	60	60	60
	Administering flumazenil intramuscularly	28	13	13	73
	Administering atropine intravenously	33	15.3	15.3	88.4
	Administering epinephrine intramuscularly	25	11.6	11.6	100
If a patient is experiencing anaphylaxis, what is the immediate priority?	Administer an epinephrine auto-injector	121	56.3	56.3	56.3
	Provide the patient with antihistamines	64	29.8	29.8	86
	Make the patient lie down and elevate their feet	23	10.7	10.7	96.7
	Give the patient water to drink	7	3.3	3.3	100
In case of anaphylactic shock, what drug is the first line of treatment?	Steorids	26	12.1	12.1	12.1
	Antihistamines	47	21.9	21.9	34
	Enipherine	139	64.7	64.7	98.6
	Insulin	3	1.4	1.4	100
Exploration					
In your opinion, what role do nurses and dental assistants play in emergency situations, and what are their responsibilities in such scenarios?	Vital signs monitoring	14	6.5	6.5	6.5
	Calling for emergency	9	4.2	4.2	10.7
	Assistance with medication administration	12	5.6	5.6	16.3
	All of above	180	83.7	83.7	100
What are the main factors that prevent you from receiving adequate training and preparation for MEs during your studies?	Lack of hands-on practice and simulation exercises in the curriculum	82	38.1	38.1	38.1
	Limited access to emergency equipment and supplies for training purposes	52	24.2	24.2	62.3
	Insufficient emphasis placed on the importance of emergency care skills	27	12.6	12.6	74.9
	Limited opportunities to learn through interprofessional collaboration	54	25.1	25.1	100

In examining specific emergency scenarios, the data indicates that 80.5% of students correctly identified the initial action when dealing with an unconscious patient as checking the patient's airway, breathing, and circulation (ABCs). This response underscores the importance placed on the primary survey in emergency care, which is vital for determining the immediate needs of the patient. Furthermore, in cases of suspected spinal injury, an overwhelming 83.3% of students prioritized immobilizing the patient's head and neck, highlighting their awareness of the critical need to prevent further injury in such scenarios.

Table 2 also explores responses to a hypothetical situation where a 45-year-old male collapses in a public place and is unresponsive. Here, 38.1% of students indicated they would start chest compressions, while 40% would call for emergency medical services (EMS). These responses reflect the dual emphasis on immediate life-saving interventions and the importance of summoning professional medical assistance.

When it comes to cardiovascular emergencies, 57.2% of students recognized that administering aspirin is the appropriate first step for a patient with a possible heart attack, demonstrating an understanding of the role of antiplatelet therapy in managing myocardial infarction. Additionally, 75.8% of students correctly identified hemoptysis as not being a sign of myocardial infarction, indicating a nuanced understanding of the clinical presentations of cardiac events.

For respiratory emergencies, the study found that 56.7% of students chose inhaled albuterol as the first-line treatment for acute asthma exacerbation, which aligns with current medical guidelines. In cases where a patient is suspected of having aspirated a foreign body, 51.2% of students would place the patient in the reverse Trendelenburg position and encourage coughing, reflecting appropriate clinical management of such situations.

In neurological emergencies, 54.4% of students identified intravenous diazepam as the first-line treatment for status epilepticus, consistent with standard emergency protocols. For stroke management, 72.6% of students would call emergency services, monitor vital signs, and provide supportive care, highlighting their awareness of the critical steps needed to manage a potential cerebrovascular accident effectively.

The overdose section of student responses reveals insightful data regarding their knowledge and actions when dealing with overdoses. The primary treatment for a patient with a suspected opioid overdose was primarily identified as intravenous naloxone, with 60.0% of students choosing this option. This highlights a strong understanding of standard emergency intervention for opioid overdose among students. 33 students (15.3%) chose intravenous atropine, indicating some confusion, as atropine is not a primary treatment for opioid overdose. Intramuscular flumazenil was chosen by 13.0%, and intramuscular epinephrine was the least commonly chosen, at 11.6%.

When addressing anaphylaxis, the priority of administering an epinephrine auto-injector was recognized by 56.3% of the students. This response indicates an adequate level of preparedness for this severe allergic reaction, although a significant proportion, 29.8%, incorrectly prioritized providing antihistamines first. Making the patient lie down and elevate their feet was correctly identified by 10.7% of students as part of the supportive measures, while giving the patient water to drink was least chosen at 3.3%.

In the scenario of anaphylactic shock, epinephrine was correctly identified as the first-line treatment by 64.7% of students, demonstrating their knowledge of emergency protocols. However, 21.9% incorrectly chose antihistamines, and a smaller percentage selected steroids (12.1%) and insulin (1.4%), indicating areas where further education might be needed.

 Overall, these responses highlight a general awareness among students of critical emergency interventions, with naloxone and epinephrine being correctly identified as primary treatments in most cases. However, the selection of less appropriate treatments by a noticeable fraction of students suggests that additional training and clarification on emergency protocols could enhance their readiness and effectiveness in real-world scenarios

## Discussion

Dental and medical students have a typically solid foundation in emergency care, according to a review of their understanding of BLS and emergency response. A strong foundation in emergency management is demonstrated by the noteworthy 78.6% of students who are conversant with BLS protocols. With an emphasis on the vital need of evaluating ABCs, the majority of students (80.5%) correctly identified the first actions for an unconscious patient. A sizable portion (83.3%) showed knowledge of the immobilization of the head and neck in cases of spinal injury. When faced with an emergency, 38.1% of students initiated chest compressions on an unresponsive patient, and 40% chose to call 911, striking a balance between rapid action and professional assistance. 75.8% of respondents correctly identified hemoptysis as unrelated to myocardial infarction (MI), and 57.2% of respondents identified aspirin as the first step for suspected heart attacks in cardiovascular emergencies. 51.2% of respondents indicated the reverse Trendelenburg position for aspiration of a foreign body in respiratory emergencies, while 56.7% of respondents selected inhaled albuterol for asthma. Regarding neurological problems, 72.6% emphasized calling emergency services and providing supportive care for stroke victims, while 54.4% identified intravenous diazepam as treatment for status epilepticus. Overall, even when students demonstrate a high level of understanding, ongoing education is essential to ensure full disaster preparedness. The current study builds upon the findings from the previous research, providing a comprehensive assessment of the knowledge and preparedness of dental and medical students in handling MEs.

While earlier studies by Mohideen et al., Jodalli and Ankola, and Gazal et al., highlighted deficiencies in students' knowledge and awareness regarding the management of MEs, our study demonstrates that students now have a generally solid foundation in emergency care, with 78.6% being conversant with BLS protocols and 80.5% correctly identifying the first actions for an unconscious patient [2,6,7]. However, the findings also suggest that there are still areas for improvement, as students exhibited varying levels of knowledge in more specific emergency scenarios, such as 75.8% correctly identifying hemoptysis as unrelated to MI, and 57.2% identifying aspirin as the first step for suspected heart attacks. This aligns with the emphasis in the previous studies by Toback and Bank and Brazil, on the importance of ongoing education and training to ensure full disaster preparedness [3,5]. By incorporating these comparative points, you can strengthen the discussion and analysis section of your dissertation, highlighting the current state of knowledge and the need for continued efforts in emergency preparedness education for dental and medical students.

Study limitations

One of the main limitations of this study is its cross-sectional design. By collecting data at a single point in time, the study provides a snapshot of the students' knowledge and preparedness but does not capture any changes or improvements that may occur over the course of their education and training. A longitudinal study design, where the students are assessed at multiple time points, would have allowed for a more comprehensive evaluation of the development of their emergency care competencies.

## Conclusions

The analysis of BLS knowledge and emergency response among dental and medical students indicates a robust understanding of fundamental emergency care. A significant majority of students demonstrated awareness of essential procedures, such as assessing airway, breathing, and circulation (ABCs), and managing spinal injuries. They also showed appropriate responses to various emergency scenarios, including cardiovascular, respiratory and neurological emergencies. Despite high levels of correct responses, the study highlights the need for ongoing training to address knowledge gaps and ensure comprehensive preparedness. Continuous education and practical experience are crucial in equipping future healthcare professionals to handle MEs effectively.
